# The effect of mother-infant skin-to-skin contact immediately after birth on exclusive breastfeeding: a systematic review and meta-analysis

**DOI:** 10.4274/jtgga.galenos.2019.2018.0138

**Published:** 2020-03-06

**Authors:** Fatemeh Zahra Karimi, Hamid Heidarian Miri, Talat Khadivzadeh, Nahid Maleki-Saghooni

**Affiliations:** 1 Nursing and Midwifery Care Research Center, Mashhad University of Medical Sciences, Mashhad, Iran; 2 Department of Midwifery, School of Nursing and Midwifery, Mashhad University Medical of Medical Sciences Mashhad, Iran; 3 Department of Epidemiology and Biostatistics, Social Determinants of Health Research Center, Mashhad University of Medical Sciences, Mashhad, Iran; 4 Ph.D. Student of Reproductive Health, School of Nursing and Midwifery, Mashhad University of Medical Sciences, Mashhad, Iran

**Keywords:** Mother-infant skin-to-skin contact, exclusive breastfeeding, systematic review, meta-analysis

## Abstract

In the new millennium, exclusive breastfeeding plays an important role in national and international policies. The effects of mother-infant skin-to-skin contact (SSC) after birth has been investigated in several studies. Given that there has been no overall estimate of this effects, the present study was conducted with the aim of investigating the effects of mother-infant SSC on the rate of exclusive breastfeeding through a systematic review and meta-analysis of randomized controlled trials. In the present study, the databases of Scopus, PubMed, Cochrane, SID, Magiran, IranDoc, and Google Scholar were searched to identify randomized controlled trials that evaluated the effects of mother-infant SSC immediately after birth on the rate of exclusive breastfeeding. The risk of bias and strength of evidence were examined according to the Cochrane Collaboration’s tool and the Grading of Recommendation, Assessment, Development, and Evaluation approach, respectively. The data analysis was performed using Stata software. To assess the publication bias and heterogeneity, Egger’s and Begg’s tests and I^2^ were used, respectively. In addition, the fixed effects model was employed to perform the meta-analysis. The heterogeneity of the factor of effects in the studies was determined as 16.2% (p<0.303). There was no publication bias among the studies included; the p values of Egger’s and Begg’s tests were 0.168 and 0.386, respectively. The effects of mother-infant SSC on exclusive breastfeeding was statistically significant [odds raito (OR)=2.19; 95% confidence interval (CI): (1.66-2); p<0.001]. The subgroup analysis results in the normal vaginal delivery group included OR=2.45 [95% CI: (1.76-3.35); p<0.001], for the cesarean delivery group the results were OR=1.44 [95% CI: (0.78-2.65); p=0.24], the results for the duration of exclusive breastfeeding as of the discharge time up to 3 months were OR=2.47 [95% CI: (1.76-3.48); p<0.001], and the results for the 3 to 6 months of exclusive breastfeeding were OR=1.71 [95% CI: (1.05-2.78); p=0.030]. The study results showed that mother-infant SSC increased the rate of exclusive breastfeeding. Therefore, this finding could be used by maternal and infant health care providers to develop evidence-based intervention programs.

## Introduction

Infants are quite vulnerable in the early stages of their life, and due to the higher speed of their growth than at other life stages, it is important that they are provided with sufficient energy, proteins, and other nutrients vital for their future health. Breastfeeding is the most ideal nutrition method at this stage. Hence, breastfeeding plays an important role in formulating national and international policies on general health, child survival, and maternal health, in the new millennium (1,2,3,4,5,6,7).

Breastfeeding benefits will be maximized if practiced exclusively in the first 6 months after birth and then continued with supplementary nutrition up to 2 years. However, the reduction in the rate of exclusive breastfeeding is one of the most prevalent and serious problems at the present time. This rate is low in many world countries, having also been declining over the past few years in our country. In Iran, according to the Ministry of Health, the rate of breastfeeding has been plunging in recent years, with the exclusive breastfeeding rate having reached 56.8% and 27.9% by the end of the 4^th^ and 6^th^ month, respectively. This fact required the Office of Research and Technology of the Ministry of Health to develop the breastfeeding promotion program as one of the country’s research priorities (1,8,9); based on this program, it is required that breastfeeding methods be developed and adopted.

Previous studies have shown that the life environment of infants before, during, and immediately after birth, the measures adopted during pregnancy and after birth, and the hospital policy affect the length of the breastfeeding period. These factors can affect the breastfeeding mechanism and the neonate’s primary sucking behaviors, thereby leading to the discontinuation of breastfeeding and a reduction in the exclusive breastfeeding rate (10,11,12); hence, they are required to be taken into account because the early hours after birth are the most ideal time for a baby to start nutritional behaviors, such as searching and sucking. During this period, most babies respond to the tactile, warm, and olfactory stimuli of their mothers’ body and become capable of sucking, and thus they start getting breastfed. Therefore, the early hours after birth are critical for the establishment and continuation of breastfeeding (1,10,13). Research has shown that the separation of the baby from the mother at birth, even for a short time, for the purpose of activities such as the evaluation of the baby, vitamin K injections, as well as the repair of an episiotomy and perineal injuries, could exert negative physiologic effects on the baby, including creating stress and increasing baby crying. As a result, the consumption of stored energy reduces the neonate’s success in initiating nutritional behaviors, thereby affecting the stimuli and necessary responses to the development of sucking skills, effective breastfeeding, and the breastfeeding duration (10,13).

The study by DiGirolamo et al. (14) showed that the delay in starting breastfeeding was the major risk factor in the premature interruption of breastfeeding. Hence, skin-to-skin contact (SSC) between the mother and the infant is recommended at the first moments after birth so as to promote breastfeeding. SSC between the mother and the infant is a method in which the naked newborn is placed in the prone position on the mother’s bare chest immediately after birth or during the first 24 hours after birth. During mother-infant SSC, the interaction between the mother and infant is enhanced, leading to an increase in the neonate’s response to the mother’s body stimulation and the development of nutritional behaviors in the baby (15,16,17).

Most studies have shown that SSC is beneficial for the mother and the baby. Nevertheless, there are conflicting studies on the relationship between mother-infant SSC and breastfeeding. Mahmood et al. (18), Gouchon et al. (19), Marín Gabriel et al. (20), and Vaidya et al. (21) reported that mother-infant SSC immediately after birth increases exclusive breastfeeding significantly. However, in the studies by Moore et al. (32) and Carfoot et al. (22), there was no significant relationship observed between mother-infant SSC and the exclusive breastfeeding rate (13).

Systematic reviews and meta-analyses are essential tools for summarizing the evidence available in a precise, accurate, and reliable manner (23). Despite the fact that several studies have been performed on the effects of mother-infant SSC on exclusive breastfeeding, the contradictory findings of these studies necessitate performing a meta-analysis that provides clear and coherent results and a comprehensive guide for policy makers and researchers. Thus, the present meta-analysis was performed with the aim of investigating the effects of mother-infant SSC immediately after birth on exclusive breastfeeding.

## Materials and Methods

In the present systematic review and meta-analysis, all studies on the effects of mother-infant SSC immediately after birth on exclusive breastfeeding were investigated using the following search terms: breastfeeding, breast feeding, lactation, or human milk; SSC, skin-to-skin mother-infant contact, SSC or kangaroo mother care (KMC) methods; KMC; exclusive breastfeeding, breastfeeding exclusivity, and breastfeeding status; and randomized clinical trials, and their Persian equivalents in the electronic databases of SID, Magiran, IranDoc, Scopus, PubMed, ISI Web of science, Cochrane, and Google Scholar from 2000 up to April 2018. In addition, a manual search was performed in the reference section of relevant trials, systematic reviews, and meta-analyses to identify trials missed by the electronic search.

**Figure 1:**
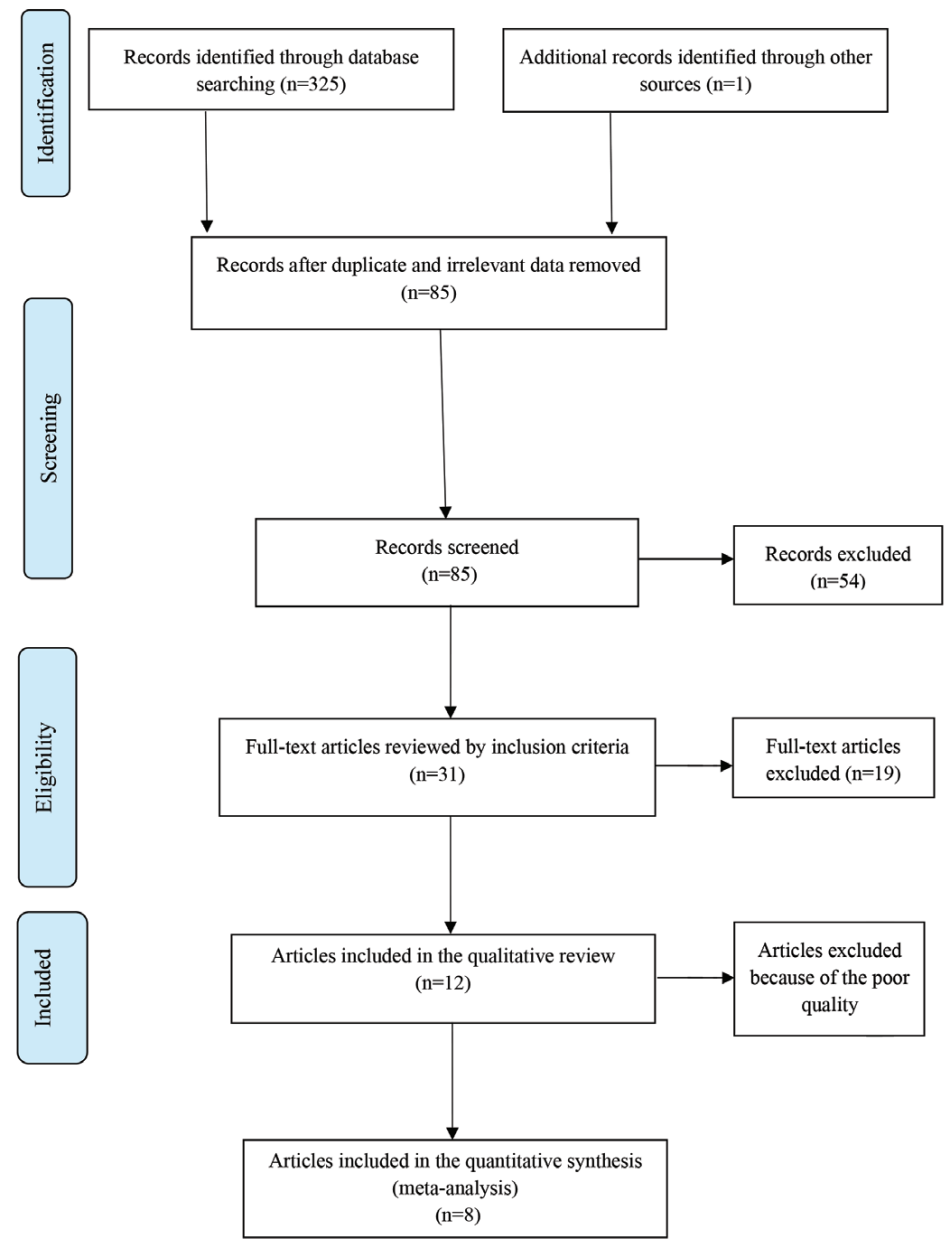
The PRISMA flowchart of the study’s selection process PRISMA: Preferred Reporting Items for Systematic Reviews and Meta-Analyses

The search and selection processes of the trials are shown using the PRISMA flowchart (Figure 1), with the PRISMA checklist used to report the meta-analysis results.

The inclusion criteria were (1) studies with an randomized-control trial (RCT) design, (2) the interventions that consisted of SSC defined as the placing of the naked neonate in the prone position on the mother’s bare chest within 10 minutes of birth, (3) the participants consisted of mothers and healthy infants between 37 to 42 weeks of pregnancy, and (4) the primary outcome was exclusive breastfeeding up to six months after birth. There was no secondary outcome included.

To study the selection, first, the abstracts and keywords of relevant articles and their eligibility were examined in view of the inclusion criteria. Secondly, the full texts of the articles were reviewed independently by two authors for eligibility and discussed until consensus was reached.

The risk of bias was examined for each study by two independent evaluators using the Cochrane Collaboration’s tool. In the event of a disagreement between the two evaluators, the issue would be resolved by a third researcher. Using the mentioned tool, 6 types of biases were assessed, including selection bias (random sequence generation and allocation concealment), performance bias (examining the blinding of participants and personnel), detection bias (the blinding of outcome assessors), attrition bias (incomplete outcome data), reporting bias (selective reporting), and other sources of biases. Based on the degree of each type of bias, the studies were assessed and reported with low, high, and uncertain risks (24,25).

To assess the overall strength of the evidence, the Grading of Recommendation, Assessment, Development, and Evaluation (GRADE) approach was adopted.

The extracted data were registered on relevant forms. The two authors extracted data from the full text of the articles independently, based on the data collection form.

The collected data included the authors’ names, publication years, study designs, sample sizes, tools, outcomes, and the risks of bias assessment. After data collection, the extracted data were assessed. The data analysis was performed using the STATA 14.1 program. The effect level was calculated as the odds ratio, with the odds of exclusive breastfeeding in the intervention group divided by that of the control group. Next, subgroup analysis was performed based on the type of delivery and duration of exclusive breastfeeding to assess heterogeneity among the studies.

I^2^ and its p-value were used to assess heterogeneity among the studies. In addition, a fixed effects model was applied to the pooled data and a meta-analysis was performed. Publication bias among the studies was assessed statistically using Egger’s and Begg’s tests, and visually using a funnel plot.

## Results

**Figure 2:**
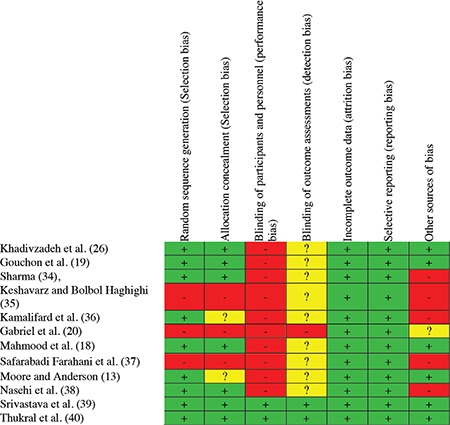
Author’s judgments of risk of bias items for each study included

**Figure 3:**
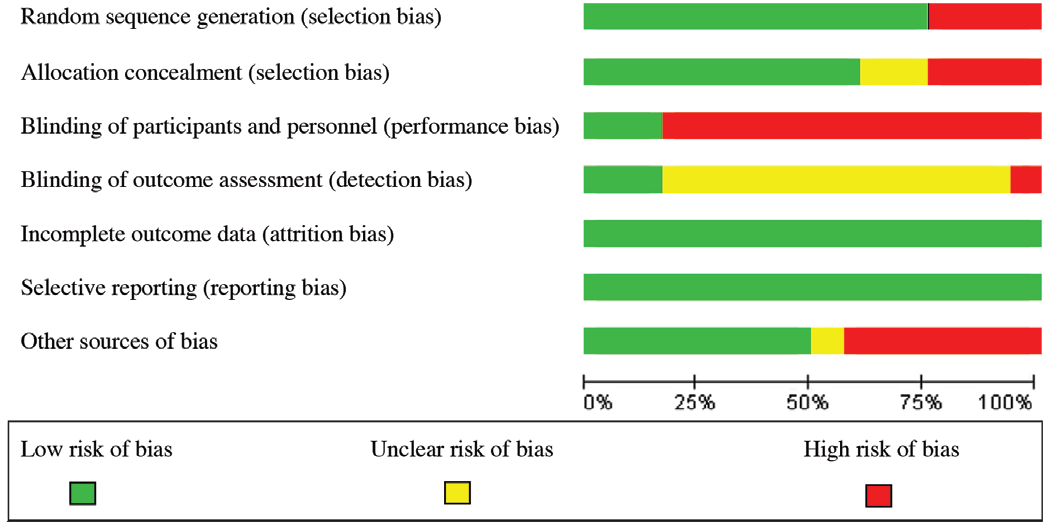
Authors’ judgments of risk of bias presented as percentages across all included studies

In the primary search, 326 articles were obtained, with a total of 12 trials that met the inclusion criteria of the study. Out of the 12 studies, 5 studies were conducted in Iran, 3 India, and the other 4 were conducted in Pakistan, Italy, the United States, and Spain. Exclusive breastfeeding was assessed by asking questions from mothers on the phone or via face-to-face interviews in most studies (n=9). The language of 6 studies was English, and 3 studies were in Persian. The data extracted from the studies included in the meta-analysis are presented in Table 1. The results of assessing the risk of bias in the studies, using the Cochrane Collaboration’s tool, are shown in Figures 2, 3.

**Figure 4:**
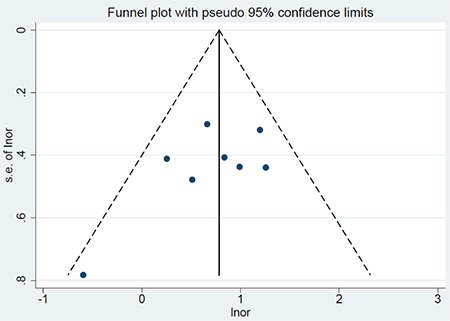
The funnel plot for the publication bias x-axis is the natural logarithm of the odds ratio and the y-axis is the standard error of the natural logarithm of odds ratio

Publication bias was assessed statistically using Egger’s and Begg’s tests. The p values of the Egger’s and Begg’s tests were 0.168 and 0.386, respectively, indicating that no publication bias existed among the studies included. The symmetric pattern of the funnel plot also confirmed the preceding statistical tests visually (Figure 4).

The heterogeneity of the measure of effects among the studies was assessed based on I^2^, which was 16.2% (p<0.303). However, a random effects model was applied to all calculations because it was assumed that some of the differences between the studies could be factual.

**Figure 5:**
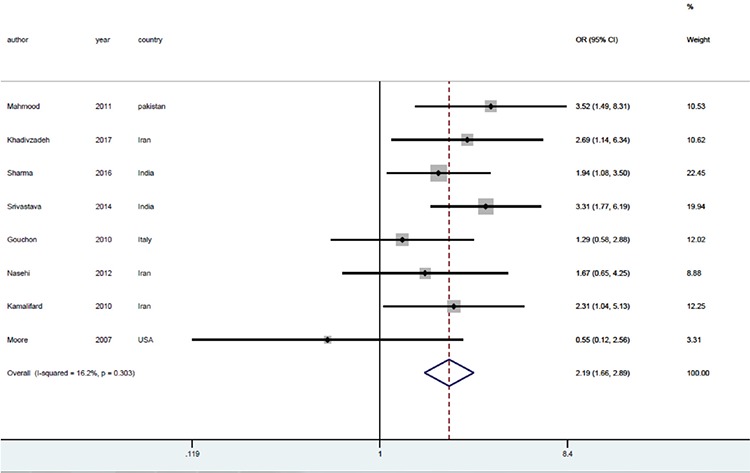
The effects of mother-infant skin-to-skin contact on exclusive breastfeeding based on the odds ratio. The horizontal lines denote the 95% CI, the Square (□) shows the point estimate (the size of the square corresponds to its weight); the diamond shows (◇) the combined overall effects of treatments. OR: Odds ratio, CI: Confidence interval

All studies were included in the meta-analysis, which resulted in an odds ratio (OR) of 2.19 with 95% confidence interval (CI) (1.66-2.89), implying that the effects of mother-infant SSC on exclusive breastfeeding were statistically significant (p<0.001). The forest plot, the odds ratio, its CI, the corresponding weight of each individual study, the pooled OR, its CI, and the I^2^ index are shown in Figure 5.

**Figure 6:**
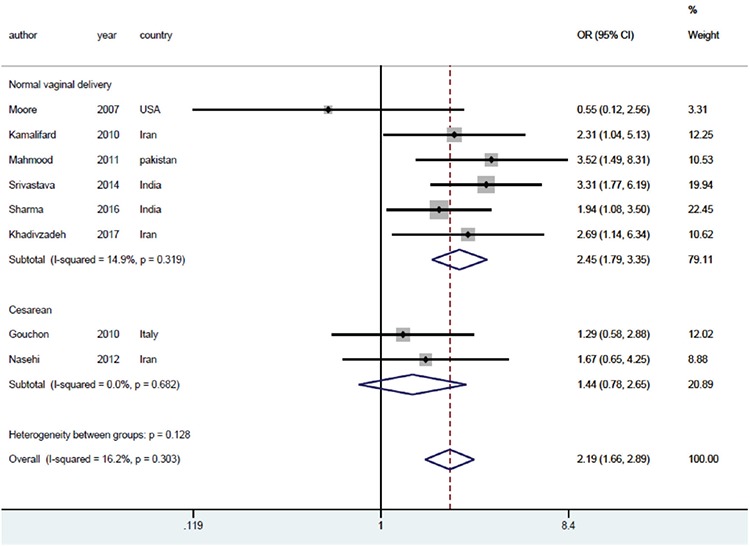
The effects of mother-infant skin-to-skin contact on exclusive breastfeeding based on the type of delivery. The horizontal lines denote the 95% CI, the square shows (□) the point estimate (the size of the square corresponds to its weight); the diamond shows (◇) the combined overall effects of treatments OR: Odds ratio, CI: Confidence interval

Next, subgroup analysis was performed based on the type of delivery and periods of exclusive breastfeeding to examine the impact of these two variables on the results (Figures 5, 6). The subgroup analysis, based on the type of delivery, resulted in lower heterogeneity among the studies in the normal vaginal delivery group (I^2^=14.9%, p=0.319), yet lower heterogeneity in the cesarean delivery group (I^2^=0.0%, p=0.682). However, the measure of the effects OR was 2.45 with a 95% CI (1.76-3.35) for the normal vaginal delivery group and 1.44 with a 95% CI (0.78-2.65) for the cesarean delivery group (Figure 6).

**Figure 7:**
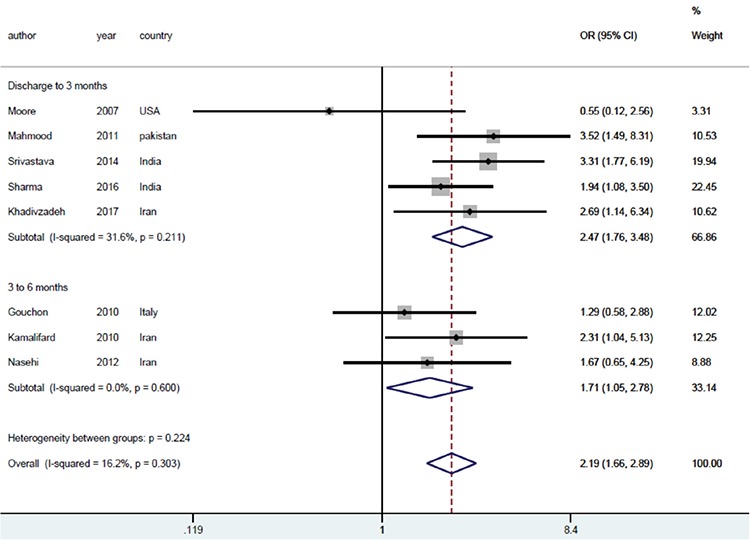
The effects of mother-infant skin-to-skin contact on exclusive breastfeeding based on the periods of exclusive breastfeeding. The horizontal lines denote the 95% CI, the Square (□) shows the point estimate (the size of the square corresponds to its weight); the diamond shows (◇) the combined overall effects of treatment OR: Odds ratio, CI: Confidence interval

The same pattern was observed when the subgroup analysis was performed based on the duration of exclusive breastfeeding (from the discharge date up to 3 months, as well as 3 to 6 months). In addition, heterogeneity increased in one group (I^2^=58.1%, p=0.26) and decreased in the other (^2^=0.0%, p=0.745). Similarly, the subgroup analysis did not change the measure of the effects significantly. The OR values were 2.47 with a 95% CI (1.76-3.48) in the first group, and 1.71 with a 95% CI (1.05-2.78) in the second group (Figure 7).

The sensitivity analysis showed that the restriction of the meta-analysis did not change the results significantly, but improved the quality of the body of evidence evaluated based on the GRADE approach (Table 2).

## Discussion

In this systematic review and meta-analysis, 12 RCTs were reviewed that had investigated the effects of mother-infant SSC on exclusive breastfeeding. The results of this meta-analysis indicated that mother-infant SSC had more statistically significant effects on exclusive breastfeeding than routine care. In the analysis of the subgroups performed based on the type of delivery and duration of exclusive breastfeeding, it was determined that mother-infant SSC had a statistically significant effect on exclusive breastfeeding.

Past research has shown that the life environment of the baby before, during, and immediately after birth, the measures adopted during pregnancy and after birth, and the hospital policy affect the breastfeeding of neonates and are strong predictive factors of the duration of exclusive breastfeeding (26,27,28). One of the midwifery interventions in hospitals is the separation of the mother from the baby for medical reasons, immediately after birth. The reasons cited by hospitals for isolating the mother from the baby include monitoring the baby quickly after birth to stabilize their physical and medical conditions, preventing mother-to-baby infection transmission, providing more time for the mother’s sleep and comfort, and assessing the baby medically (26). Research in this field showed that the separation of the mother from the neonate after birth could exert adverse effects on the mother and the baby; this could result in reducing the interaction between the mother and the baby, making the baby fail to display nutritional behaviors, causing delays in lactation, reducing the mother’s self-esteem and self-efficacy in relation to breastfeeding, impairing breastfeeding, reducing the breastfeeding duration, and finally leading to the use of other foods (1,27,29,30).

To solve the problem, skin contact between the mother and the neonate was introduced, in which the baby was placed on their abdomen in chest-to-chest contact with the mother. The principle of skin contact between the mother and the baby is derived from studies on animals. It has been demonstrated in animal studies that some of the instinctive behaviors seen in neonates are essential for their survival, with the neonates’ survival being dependent on close contact with the mother. From the standpoint of ethologists, the early postnatal hours when the fetus is transmitted to the ectopic, undergoing rapid and profound physiologic changes, are critical in the neonate’s adaptation in a short time so as to survive. Ethologists believe that the early hours after birth are ideal for starting baby’s nutritional behaviors, such as searching and sucking, and are sensitive and critical times for breastfeeding because most babies respond to tactile, warm, and olfactory stimuli of the mother’s body. Hence, the separation of the mother and baby immediately after birth could lead to the discontinuation of such instinctive behaviors (31). During SSC, the interaction between the mother and the baby increases, thereby leading to the development of nutritional behaviors that result in the baby’s sucking on the mother’s breasts and being nourished. It has been reported in relevant research that breastfeeding started immediately after birth ensures greater continuity. Postnatal skin contact between the mother and the baby is one of the measures recommended by the World Health Organization and UNICEF to increase the rate of breastfeeding because of the importance of this issue and based on the existing evidence (1,13,26,31).

In the same vein, Moore et al. (32) study showed that postnatal skin contact between the mother and the neonate exerted beneficial effects on exclusive breastfeeding. Although this study confirmed the beneficial effects for the mother and neonate, it had some limitations that justified the need for the current study. Some of the limitations included the examining of several different variables in the study that reduced the accuracy of the search, and the non-inclusion of all eligible studies by the data extracted, especially the ones published in Persian, which resulted in a decrease in the input of the final sample size into the meta-analysis and the affect analysis.

Forster and McLachlan (29), in a narrative review, stated that skin contact between the mother and neonate was one of the ways of boosting breastfeeding. In fact, the study by Foster was merely a literature review. By contrast, the present study is a systematic and meta-analytic review; this type of study provides the best evidence for judging the impact of interventions in medicine and their use in clinical settings.

Given that exclusive breastfeeding can provide specific nutrients, both in quality and quantity for the neonate by the end of the 6^th^ month after birth, it provides all the neonate’s nutritional needs necessary for normal development. In addition, given that the reduction in the exclusive breastfeeding rate is one of the major public health problems (33), the need for identifying ways of establishing and maintaining exclusive breastfeeding is evident. In this regard, according to the results of the present study, the postnatal skin contact of the mother and neonate could increase the exclusive breastfeeding rate. It is suggested that contact between the mother and baby be adopted as a care method by maternal and child health care providers, such as midwives, doctors, and students responsible in childbirth (34,35,36,37,38,39,40).

One of the strengths of the present study is that it is the first systematic and meta-analytic review study in Iran to investigate the effects of postnatal mother-neonate skin contact on exclusive breastfeeding. One of the limitations of this study is the quality of the studies included in terms of their methodologies. Thus, it is recommended that clinical trials be performed using more qualitative methodologies to obtain more positive evidence.

## Conclusion

The articles reviewed in this systematic review and meta-analysis showed that mother-infant SSC increased the exclusive breastfeeding rate. Thus, contact provides the best postnatal care for neonates. In spite of the evidence provided and the benefits of close postnatal contact between the mother and baby, this is not practiced satisfactorily in Iran. In addition, in many cases, the mother and neonate are separated after birth to perform conventional hospital practices, which seems to play an important role in causing lactation disorders. Perhaps this is the reason why, despite the benefits of exclusive breastfeeding in the first 6 months after birth, exclusive breastfeeding rates have declined in our country over the past few years. Thus, the results of the present study could be used in evidence-based decision-making by policy makers and service providers in the field of maternal and child health care as a guide for increasing the exclusive breastfeeding rate.

## Figures and Tables

**Table 1:**
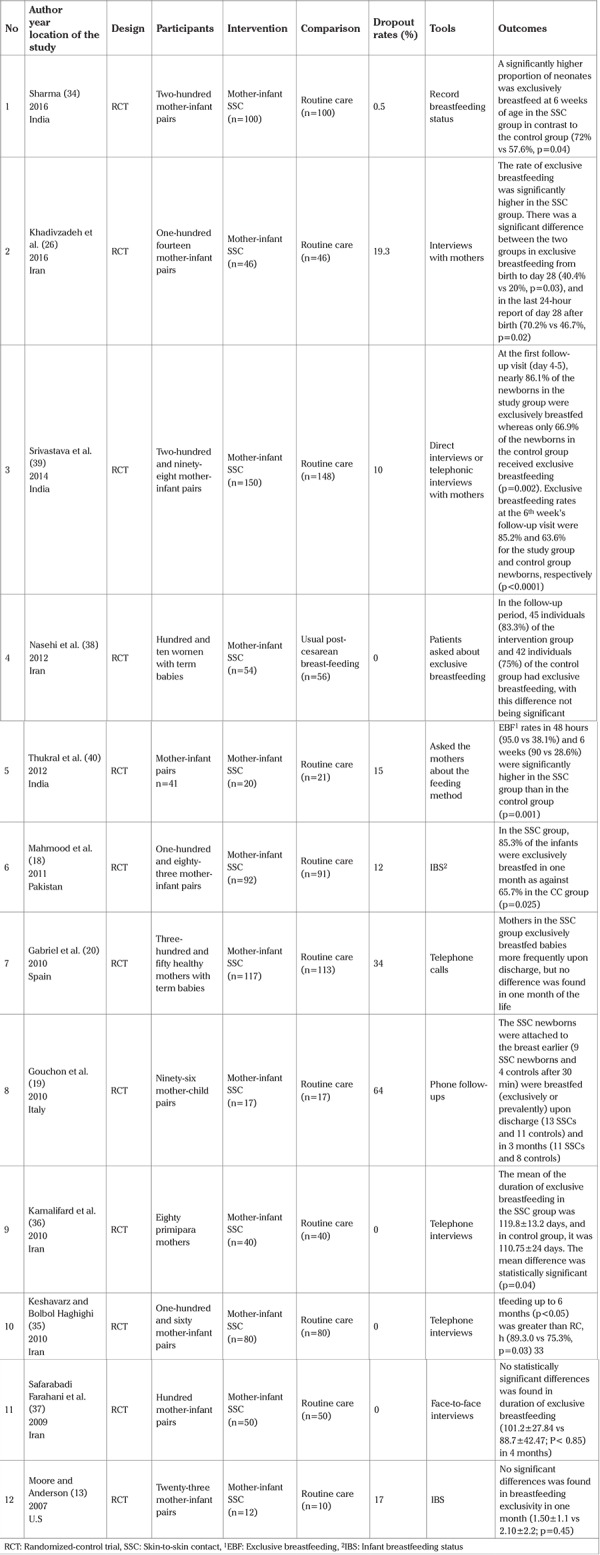
Features of 12 clinical trials included in study

**Table 2:**
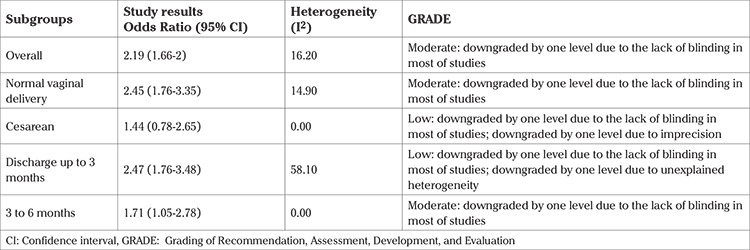
The Grading of Recommendation, Assessment, Development, and Evaluation evidence profile for the effects of mother-infant skin-to-skin contact immediately after birth on exclusive breastfeeding for the type of delivery and duration of exclusive breastfeeding

## References

[1] Lawrence RA, Lawrence RM (2005.). Breastfeeding, A guide for the medical profession. 6th ed. Philadelphia: Elsevier, Mosby.

[2] Sriraman NK, Evans AE, Lawrence R, Noble L;, Academy of Breastfeeding Medicine’s Board of Directors (2018). Academy of Breastfeeding Medicine’s 2017 position statement on informal breast milk sharing for the term healthy infant. Breastfeed Med.

[3] Saghooni NM, Barez MA, Moeindarbari S, Karimi FZ (2017). Investigating the breastfeeding self-efficacy and its related factors in primiparous breastfeeding mothers. Int J Pediatr.

[4] Anbaran ZK, Baghdari N, Pourshirazi M, Karimi FZ, Rezvanifard M, Mazlom SR (2015). Postpartum sexual function in women and infant feeding methods. J Pak Med Assoc.

[5] Gartner LM, Morton J, Lawrence RA, Naylor AJ, O’Hare D, Schanler RJ, et al (2005). Breastfeeding and the use of human milk. Pediatrics.

[6] WHO, UNICEF, CDD. Participants manual part three Sessions 1-9. Accessed Mar 26, 2007. Available at:.

[7] Anbaran ZK, Baghdari N, Sadeghi Sahebzad E, Moradi M, Karimi FZ (2016). Comparing infant nutrition in wanted and unwanted pregnancies. Int J Pediatr.

[8] Ministry of Health and Medical Education, Deputy of Health. Research project of the new system of monitoring and evaluation of reproductive health services IEMS. Available at:.

[9] No authors listed Ministry of Health and Medical Education, Dean of Research and Technology. The priority of breastfeeding research. [Correspondence].

[10] Karimi A, Bagheri S, Khadivzadeh T, Mirzaii Najmabadi K (2014). The effect of an interventional program, based on the theory of ethology, on infant breastfeeding competence. Iranian Journal of Neonatology.

[11] Academy of Breastfeeding Medicine, Protocol Committee. Clinical Protocol #5: Peripartum breastfeeding management for the healthy mother and infant at term. 2003. Accessed Oct 7, 2006. Available at:.

[12] Moreland J, Coombs J (2000). Promoting and supporting breast-feeding. Am FAM Physician.

[13] Moore ER, Anderson GC (2007). Randomized controlled trial of very early mother-infant skin-to-skin contact and breastfeeding status. J Midwifery Womens Health.

[14] DiGirolamo AM, Grummer-Strawn LM, Fein S (2001). Maternity care practices: implications for breastfeeding. Birth.

[15] The WHO Reproductive Health Library. Early skin-to-skin contact for mothers and their healthy newborn infants. 2006. Accessed 10Aug, 2006. Available at:.

[16] Dabrowski GA (2007). Skin to skin contact in, giving birth back to mothers and babies. Nurs Womens Health.

[17] Karimi FZ, Khadivzadeh T, Saeidi M, Bagheri S (2016). The Effect of Kangaroo mother care immediately after delivery on mother-infant attachment 3 monthsafter delivery. Int J Pediatr.

[18] Mahmood I, Jamal M, Khan N (2011). Effect of mother-infant early skin-to-skin contact on breastfeeding status: a randomized controlled trial. J Coll Physicians Surg Pak.

[19] Gouchon S, Gregori D, Picotto A, Patrucco G, Nangeroni M, Di Giulio P (2010). Skin-to-skin contact after cesarean delivery: an experimental study. Nurs Res.

[20] Marín Gabriel MA, Llana Martín I, López Escobar A, Fernández Villalba E, Romero Blanco I, Touza Pol P (2010). Randomized controlled trial of early skin-to-skin contact: effects on the mother and the newborn. Acta Paediatr.

[21] Vaidya K, Sharma A, Dhungel S (2005). Effect of early mother-baby close contact over the duration of exclusive breastfeeding. Nepal Med Coll J.

[22] Carfoot S, Williamson P, Dickson R (2005). A randomised controlled trial in the north of England examining the effects of skin-to-skin care on breast feeding. Midwifery.

[23] Liberati A, Altman DG, Tetzlaff J, Mulrow C, Gøtzsche PC, Ioannidis JP, et al (2009). The PRISMA statement for reporting systematic reviews and meta-analyses of studies that evaluate healthcare interventions: explanation and elaboration. BMJ.

[24] Gopalakrishnan S, Ganeshkumar P (2013). Systematic reviews and meta-analysis: understanding the best evidence in primary healthcare. J Family Med Prim Care.

[25] Higgins JP, Green S (2011.). Cochrane handbook for systematic reviews of interventions version 5.1. 0. New York: The Cochrane Collaboration.

[26] Khadivzadeh T, Karimi FZ, Tara F, Bagheri S (2016). The Effect of postpartum mother-infant skin-to-skin contact on exclusive breastfeeding in neonatal period: A randomized controlled trial. Int J Pediatr.

[27] Walke M (2002.). Core curriculum for lactation consultant practice. Sudbury: Jones and Bartlett.

[28] Alikasifoglu M, Erginoz E, Gur ET, Baltas Z, Beker B, Arvas A (2001). Factors influencing the duration of exclusive breastfeeding in a group of Turkish women. J Hum Lact.

[29] Forster DA, McLachlan HL (2007). Breastfeeding initiation and birth setting practices: A review of the literature. J Midwifery Womens Health.

[30] Karimi FZ, Bagheri S, Tara F, Khadivzadeh T, Mercer SMM (2014). Effect of kangaroo mother care on breastfeeding self-efficacy in primiparous women, 3 months after child birth. The Iranian Journal of Obstetrics, Gynecology and Infertility.

[31] WHO Library Cataloguing-in-Publication Data. Action plan for healthy newborn infants in the Western Pacific Region (2014–2020). 2014. Accessed 28 Apr, 2018. Available at:.

[32] Moore ER, Bergman N, Anderson GC, Medley N (2016). Early skin-to-skin contact for mothers and their healthy newborn infants. Cochrane Database Syst Rev.

[33] World Health Organization, UNICEF, Global Nutrition Targets 2025: Breastfeeding policy brief. 2014. Accessed 28 Apr, 2018.

[34] Sharma A (2016). Efficacy of early skin-to-skin contact on the rate of exclusive breastfeeding in term neonates: a randomized controlled trial. Afr Health Sci.

[35] Keshavarz M, Bolbol Haghighi N (2010). Effects of Kangaroo mother care on duration of exclusive breastfeeding and feeding pattern in neonates of mothers who delivered by cesarean section. MEDICAL SCIENCES.

[36] Kamalifard M, Heydarzadeh M, Ghogazadeh M, Mohammadi M (2010). The effect of kanagaroo mother care on exclusive breastfeeding in nuliparous. Nursing & Midwifery Journal.

[37] Safarabadi Farahani T, Ali Akbar M, Taavoni S, Haghani H (2009). The Effect of Kangaroo Contact on Duration of Exclusive Breastfeeding and Success of Lactation among Primiparous Women at Shahid Akbar-Abadi Hospital in Tehran. IJN.

[38] Nasehi MM, Farhadi R, Ghaffari V, Ghaffari-Charati M (2012). The effect of early breastfeeding after cesarean section on the success of exclusive breastfeeding. HealthMED.

[39] Srivastava S, Gupta A, Bhatnagar A, Dutta S (2014). Effect of very early skin to skin contact on success at breastfeeding and preventing early hypothermia in neonates. Indian J Public Health.

[40] Thukral A, Sankar MJ, Agarwal R, Gupta N, Deorari AK, Paul VK (2012). Early skin-to-skin contact and breast-feeding behavior in term neonates: a randomized controlled trial. Neonatology.

